# Capturing spontaneous interactivity: a multi-measure approach to analyzing the dynamics of interpersonal coordination in dance improvisation

**DOI:** 10.3389/fpsyg.2024.1465595

**Published:** 2024-11-05

**Authors:** Paige M. Whitehead, Hanne De Jaegher, Ivani Santana, Rebecca M. Todd, Stefanie Blain-Moraes

**Affiliations:** ^1^Department of Psychology, University of British Columbia, Vancouver, BC, Canada; ^2^Department of Psychology, University of Sussex, Brighton, United Kingdom; ^3^Department of Performing Arts, Federal University of Rio de Janeiro, Rio de Janeiro, Brazil; ^4^Djavad Mowafaghian Centre for Brain Health, University of British Columbia, Vancouver, BC, Canada; ^5^School of Physical and Occupational Therapy, McGill University, Montreal, QC, Canada

**Keywords:** movement, symbolic transfer entropy, social interaction, interactional synchrony, coordination dynamics, video analysis, pose estimation

## Abstract

**Introduction:**

Interpersonal coordination is widely acknowledged as critical to relating with, connecting to, and understanding others, but the underlying mechanisms of this phenomenon are poorly understood. Dance—particularly improvised dance—offers a valuable paradigm for investigating the dynamics of interpersonal coordination due to its inherent ability to connect us. However, conventional approaches to studying coordination often fail to capture the co-creative spontaneity that is intrinsic to such interactions.

**Methods:**

This study combined multiple measures of interpersonal coordination to detect moments of high coordination between two freely improvising dancers. We applied maximum correlation vectors, normalized Symbolic Transfer Entropy (NSTE), and surveys to analyze the time-varying dynamics of similarity in movement speeds, directed influence, and subjective perception of dancers engaged in an improvisation task.

**Results:**

This multi-measure approach offers a means of capturing the interplay between different dimensions of interpersonal coordination.

**Discussion:**

This approach may be used to understand the underlying mechanisms of co-creative social interactions in improvised dance and other forms of spontaneous interactivity.

## 1 Introduction

As an art grounded in the kinesthetic, dance emerges from movement and enables humans to relate to each other through deeply embodied and creative attunement. This intrinsic quality positions dance as a natural laboratory to explore the complexities of social interactions. Central to this exploration is the role of coordination in interpersonal interactions. Through coordination, humans engage in co-regulation, where interaction partners collectively adjust their patterns of verbal and nonverbal activity to facilitate communication. The role of coordination in human interaction is supported by extensive literature that illustrates the relationship between interpersonal coordination and various intra- and interpersonal markers of social bonding and prosocial behavior (for reviews see Hu et al., 2022; Mogan et al., 2017; Rennung and Göritz, 2016; Vicaria and Dickens, 2016). Several dance studies have further corroborated these findings, demonstrating that high levels of interpersonal coordination enhances empathetic ability and cooperation (Koehne et al., 2016; Reddish et al., 2013). In light of the unique relational power of embodied creative movement, dance offers a privileged medium for exploring the interactional process of coordination that underpins our socio-emotional experiences of relating to others.

Although all dance is inherently relational, some traditions of movement embrace greater degrees of spontaneous collaboration. In freely improvised group or partnered dance, the boundaries between moving and being moved are blurred, and the level of collective dynamics is especially rich as dancers creatively explore the relations of their movement quality, spatial organization, and temporal alignment. In free improvisation dance, the collective of movers are afforded and experience a state of collective agency, a state which is often accompanied by a profound feeling of togetherness (Himberg et al., 2018). In this way, Himberg et al. (2018) argue improvisation can be understood as fostering a particular quality of relation between individuals that shifts the attribution of agency from “I” toward “We.” Many quotidian interactions unfold without an explicit direction or objective and evolve through a similar dynamic of co-creation and mutual adaptation. The dynamic of mutual adaptation and regulation through movement coordination renders dance improvisation a valuable paradigm for assessing co-creative social interactions that extend beyond this particular art form into the spontaneous interactivity that pervades daily life.

Techniques for capturing spontaneous interactivity and interactional coordination are notably scarce. The majority of previous studies on interpersonal coordination focus on non-dance activities (e.g., finger-tapping tasks) (see Repp, 2005; Repp and Yi-Huang, 2013). Among the studies that have explored interpersonal coordination within dance, many have employed observational methods such as microanalysis to evaluate coordination (Evola and Skubisz, 2019; Torrents et al., 2018) or have quantitatively assessed coordination in dance contexts that either involved choreography (Brown and Meulenbroek, 2016; Crone et al., 2021; Washburn et al., 2014) or relied on an external rhythm or tempo guide (Boker and Rotondo, 2002; Ellamil et al., 2016; Reddish et al., 2013; Solberg and Jensenius, 2019). As such, existing tools for quantitatively measuring movement coordination in interaction have predominantly focused on measuring alignment in oscillatory behavior, for example, amongst dancers at night clubs (Ellamil et al., 2016; Reddish et al., 2013), or tracking the body’s overall quantity of movement (Boker and Rotondo, 2002; Himberg et al., 2018). These tools are often undergirded by a normative approach and assume that interactional movements will conform to certain expected dynamics. This approach is effective in capturing interaction guided by an external tempo or goal-oriented joint actions, such as choreography, because they allow interactors to anticipate something about the future of their interaction. However, freely improvised dance presents a distinct, and characteristically spontaneous, interactional paradigm that is not predicated on an external coordinating source. Consequently, many conventional tools for measuring interactional features of dance cannot capture the dynamics of mutual attunement that are essential to the spontaneous interactivity of free improvisation. Furthermore, it is important to capture *multiple* levels of interpersonal organization, including collective-level variables such as interpersonal coordination, individual-level dynamics that relate to mutual attunement, and the relationships between these levels. Thus, the objective of this study is to evaluate approaches to measuring moments of high coordination between two freely improvising dancers and demonstrate the value of this combination of measures as a framework for investigating interactions through multiple levels of description. Specifically, we aimed to investigate the following three research questions:

1. How accurately does measuring temporal alignment of motor behavior capture moments of high interpersonal coordination?
2. Can measuring the direction of interactivity between dancers inform our interpretation of interpersonal coordination by providing insight into the dynamics of mutual attunement?
3. What is the relationship between detected moments of high coordination and the relational dimensions of the dancers’ experiences of moving together?

## 2 Methods

### 2.1 Participants

Dancers with at least 5 years of previous improvisation experience and who were at least 19 years of age were recruited for this study through local dance organizations, collectives, and companies. Ten individuals were included in the study and partnered into five unique dyads. Pairs were already acquainted with each other in four out of the five dyads. One of the participants in the unacquainted dyad was the first author, as the original participant had to cancel on short notice (PW met the eligibility criteria). All participants completed the full session and were remunerated accordingly. This study was approved by the University of British Columbia Behavioural Research Ethics Board and written consent was obtained from all participants prior to commencing the session.

### 2.2 Experimental design

This is an embedded mixed-methods study design (Creswell et al., 2003). We first identify moments of high and low coordination between dancers using a novel quantitative approach. We then embed qualitative descriptions of the directional relationships and the subjective experience of these moments to provide complementary levels of description of intersubjectivity. Experimental sessions were divided into the following three phases: (1) a dyadic improvisation task, (2) the participants’ independent review and annotation of the recorded improvisation, and (3) a semi-guided group interview between participants and the first author (interview data not analyzed in this manuscript). The sessions took place in Vancouver, Canada at one of three dance studios.

### 2.3 Procedure

#### 2.3.1 Setup

**Figure 1:**
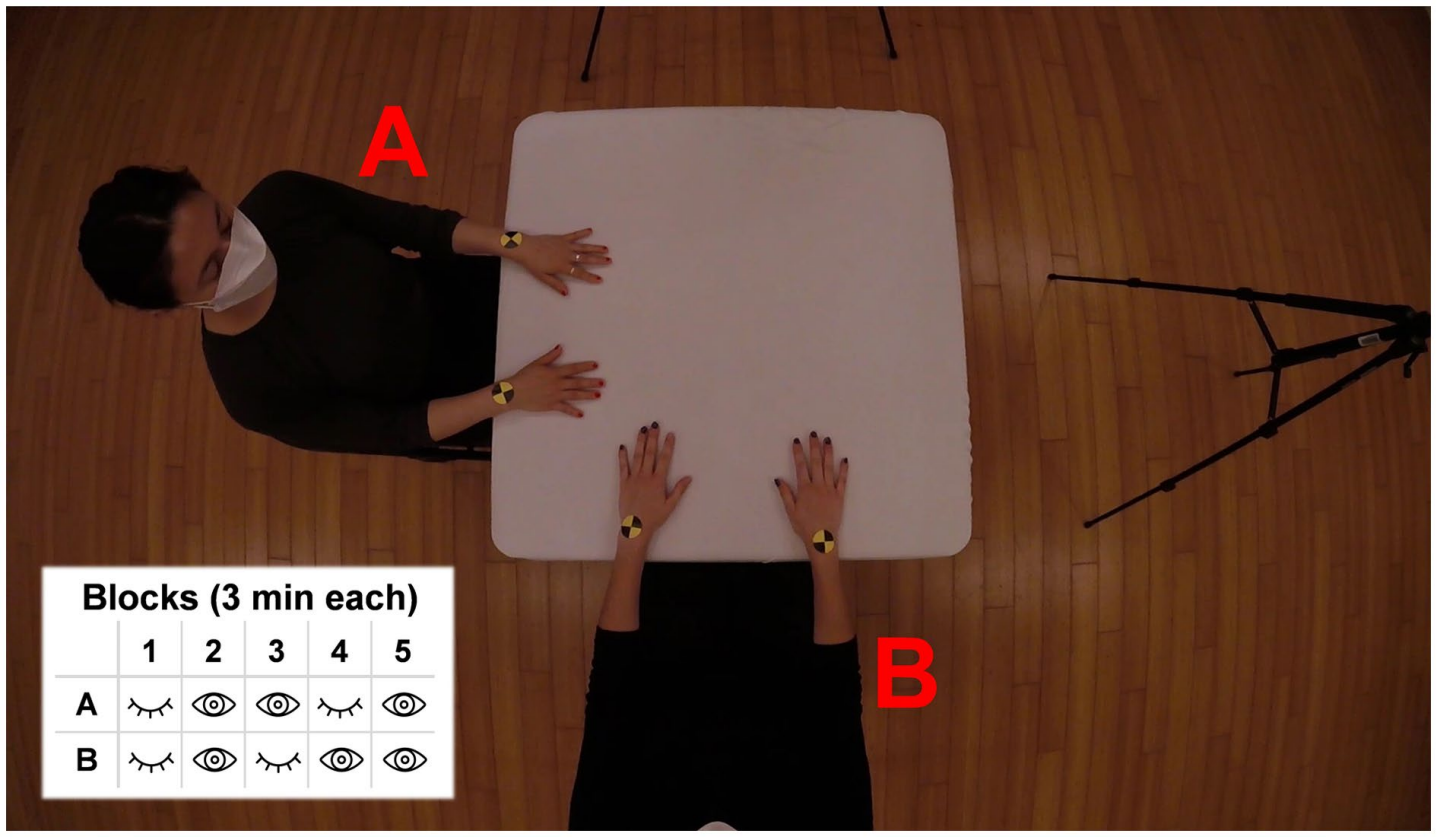
Experimental setup. The participants, labeled here with their respective referents **(A,B)** are seated perpendicular to one another at a square table. Markers (yellow and black stickers) are placed on their hands for post-experiment pose estimation using DeepLabCut (open-source pose estimation software). Improvisation task structure is included in bottom left.

Sessions began with a brief warmup led by one of the researchers. The warmup was scripted to maintain consistency across sessions and guided the participants through a body scan with gentle joint mobilizations. After the warmup, the participants were randomly assigned referents (i.e., they were told they were either dancer “A” or dancer “B”) and were seated perpendicularly to one another at a table (table dimensions: 85 × 85 cm), positioned according to their referents in preparation for the improvisation task (Figure 1). Stickers were then placed on the tops of each wrist of each participant (see Figure 1) to facilitate the extraction of spatial coordinate data from the video recordings. A video camera (GoPro Hero 4 with a frame rate of 60 Hz and a resolution of 1920 by 1080) was mounted on a microphone stand that extended over the table to obtain an aerial view of the participants and table surface.

Participants were then read a statement outlining the parameters of the improvisation task. Participants were told that they must keep their hands in contact with the table surface, as if magnetized, for the duration of the improvisation. Importantly, participants were *not* instructed to try and coordinate with their partner. They were then allowed to ask any clarification questions. Once participants were clear on the task, each completed a two-minute calibration during which they improvised independently for 1 minute with their eyes closed and 1 minute with their eyes open. This calibration period allowed participants to get acquainted with the guidelines of the task and provided an opportunity to ensure that all equipment was functioning.

#### 2.3.2 Improvisation task

The improvisation task consisted of a 15 minute seated improvisation between the two participants (Santana et al., 2021). The improvisation was divided into five 3-min blocks in which perceptual conditions were varied: we manipulated visual information available to participants to optimize our chances of eliciting wide variability in the levels of coordination between dancers (Koul et al., 2023a; Koul et al., 2023b; Miyata et al., 2017). In Block 1, both participants had eyes closed; Block 2, both participants had their eyes open; Block 3, participant A had eyes open, participant B had eyes closed; Block 4, participant A had eyes closed, participant B had eyes open; Block 5, both participants had eyes open (Figure 1). Instructions to participants to either open or close their eyes were given by a voice recording (e.g., “A close, B close”). Apart from these instructions, the improvisation was conducted in silence. Although the perceptual conditions varied, no instructions were provided to the dancers about coordination, nor the lead/follow roles that they should take should coordination emerge.

#### 2.3.3 Segmentation and commentary

**Figure 2:**
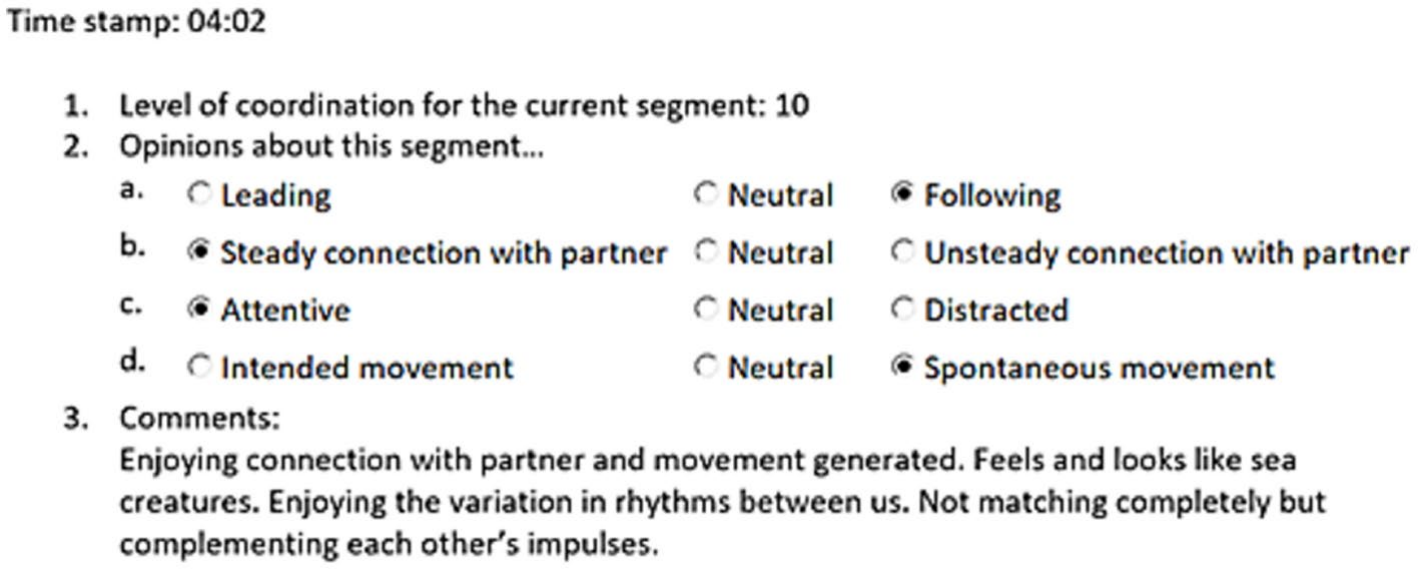
Illustration of the segmentation and commentary form structure and the data types collected from each segment.

After the improvisation, participants reviewed the video footage and marked moments in which they felt there was a transition in the interaction with their partner (Chang, 2018) (e.g., a shift in their subjective feeling of connection, a shift in the dynamic of the movement). These identified moments marked the boundary of “segments.” For each segment, participants (1) rated coordination on a 10-point scale; (2) made four binary selections about their perspective of the interaction; and (3) provided a free response commentary (see Figure 2).

### 2.4 Data extraction

**Figure 3:**
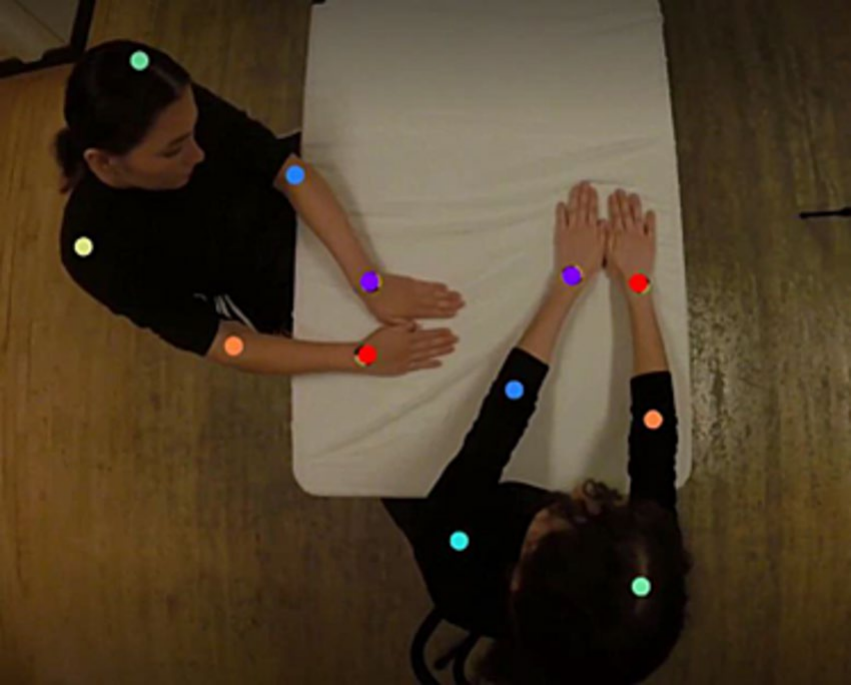
Demonstration of DeepLabCut pose estimation of user-defined body parts. Note that multiple body parts were labeled (as seen in this figure) to train a robust neural network, but only the hand data was used for analysis.

To extract kinematic data from videos, we conducted body part tracking using DeepLabCut (version 2.2.3) (Mathis et al., 2018; Nath et al., 2019) (Figure 3). We tracked both participants’ hands using the markers as a precise labeling point. Because videos were captured from an aerial perspective, spatial coordinates gave the location of each hand in two dimensions (*x*, *y*) for each video frame. Note that multiple body parts were labeled (as seen in Figure 3) to train a robust neural network, but only the hand data was used for analysis. Over-labeling is a practice recommended by DeepLabCut to improve the tracking performance of the neural network.

We labeled 360 frames taken from seven videos (95% used for training, 5% for testing). We used a ResNet101-based neural network with the system’s default parameters for 100,000 training iterations, with a resulting test error of 7.84 pixels (image size 1920 by 1080 pixels). The choice of ResNet101 was based on DeepLabCut’s recommendation for complex human activities, such as “multiple humans dancing” (Model Training Tips and Tricks—DeepLabCut, 2024). Additionally, DeepLabCut’s authors advise most users with basic proficiency in computational methods to use the default parameters, which we followed for model training. We then used a p-cutoff of 0.6 to condition the x and y coordinates for future analysis. This trained network was used to analyze and extract spatial coordinate data from video footage collected during each session. Spatial coordinate predictions for each session were filtered using DeepLabCut’s built-in median filter.

### 2.5 Data analysis

#### 2.5.1 Rating moments of high and low coordination

All recordings were coded by human raters to identify “moments” of high and low interpersonal coordination. Raters were blind to the data, including the conditions of the block. Raters were provided a definition of interpersonal coordination as the “temporal alignment of hand marker movement.” The terminology “temporal alignment” was chosen to deter raters from interpreting “interpersonal coordination” as “doing the same movement” and instead call their attention to the similarity in speed dynamics of the dancers’ movement. Notably, under this operational definition, instances of joint stillness are considered high coordination. High or low coordination needed to be sustained for at least 10 s to be considered a “moment.” The first author independently reviewed and coded the recordings, and over 50% of the moments were subsequently coded by four other raters. The coding guide was reviewed and discussed amongst all raters, and revised through iterations until all raters applied it consistently, and the inter-rater reliability reached substantial agreement.

#### 2.5.2 Movement analysis

The first research question of this study is: how accurately does measuring temporal alignment of motor behavior capture moments of high interpersonal coordination? To address this question, we developed an algorithm that calculated a novel metric of interpersonal coordination between the hands of the dancers and automatically classified the dynamic variation of this metric as a high or low coordination moment.

All movement analyses were conducted in MATLAB R2023b. Spatial coordinates (*x*, *y*) from DeepLabCut corresponding to the participants’ hands (markers) were imported into MATLAB and used to compute speed time series as follows:


$$\mathrm{spee}d_{f-1}=\frac{\sqrt{{\left(x_{f}-x_{f-1}\right)}^{2}+{\left(y_{f}-y_{f-1}\right)}^{2}}}{t},f>1$$

Where the numerator represents the distance traveled in 2D coordinate space since the previous frame (*f*-1), and the denominator represents the amount of time passed since the previous frame. This yielded a speed time series of length *n*-1 (where *n* is the total number of frames).

Speed time series were pre-processed in three steps. First, frames from any time series that was not exactly 54,000 frames (sampling rate of 60 frames/s × 15 min) were trimmed from the end of the series. Second, outliers for each session were removed (percentage of data removed: *M* = 1.43%, *SD* = 0.37%) and replaced with interpolated values. Outliers were detected as values greater than three local standard deviations away from the local mean over a 1-s window. Outlying values were then linearly interpolated (1-D interpolation). Finally, the resulting time series were smoothed using a moving mean filter (window = 0.5 s).

We then calculated a metric of interpersonal coordination over time—the maximum correlation vector (MCV)—defined by the maximum correlation of between-participant hand pairings. This measure was chosen to assign equal importance to all forms of hand coordination (i.e., independent of the number of hands involved in the coordination). The similarity in speed between participants’ movements (i.e., temporal alignment) was measured with rolling-window Spearman correlations (window = 5 s) on each between-participant time series pairing within a session (i.e., A left to B left; A left to B right; A right to B left; A right to B right). From this, four correlation vectors were produced for each session: CV_ALEFT → BLEFT_(t); CV_ALEFT → BRIGHT_(t); CV_ARIGHT → BLEFT_(t); CV_ARIGHT → BRIGHT_(t). The MCV was then calculated by selecting the maximum correlation coefficient across all hand pairings for each point in time.


$$MCV\left(t\right)=\max_{t}\{\begin{array}{c} CV_{\mathrm{ALEFT}\to \mathrm{BLEFT}}\left(t\right)\\ CV_{\mathrm{ALEFT}\to \mathrm{BRIGHT}}\left(t\right)\\ \begin{array}{c} CV_{\mathrm{ARIGHT}\to \mathrm{BLEFT}}\left(t\right)\\ CV_{\mathrm{ARIGHT}\to \mathrm{BRIGHT}}\left(t\right) \end{array} \end{array}$$

To control for spurious correlations in the data, we conducted a permutation-like test based on data sliding to assess the significance of observed correlations within each moment (Moulder et al., 2018). This involved breaking the time-dependent structure of one participant’s data, *X*, by selecting a random cut point and swapping the segments of data about the cut point (i.e., “sliding” the data) to create *X^s^*. The shuffled time series *X^s^* was then correlated with the other participant’s unshuffled time series *Y* using rolling-window Spearman correlations. Data sliding was repeated 1,000 times within each moment and significance thresholds were obtained from the 95th percentiles of the shuffled correlation distributions. The significance threshold for each rated moment is represented visually along with the MCV to enable time-resolved comparisons of the significance of the hand coordination.

We then assessed the accuracy of MCV in detecting moments of high coordination. For each rated moment of high or low coordination, we calculated a weighted summation of the values of MCV above and below threshold *y*. Evidence for low coordination was assessed using the area under the curve between *y* and MCV for all values of MCV below *y*, and evidence for high coordination was assessed using the area under the curve between MCV and *y* for all values of MCV above *y,* where *y* = 0.5. Moments were classified as high or low coordination based on the metric with the larger evidence. The predictive accuracy of our binary classification algorithm was then assessed with a confusion matrix. Ground truth was human ratings of the videos, and predicted classifications were the labels assigned by the classification algorithm. From this matrix we computed standard descriptive performance evaluation measures such as overall accuracy, precision, and sensitivity.

#### 2.5.3 Normalized symbolic transfer entropy

In our second research question, we ask if measuring the direction of interactivity between dancers can inform our interpretation of interpersonal coordination by providing insight into the dynamics of mutual attunement. To answer this, we used normalized Symbolic Transfer Entropy (NSTE) to estimate the direction and magnitude of information transfer between two kinematic source signals—in this case, the dancers’ hands. Transfer entropy (TE) is a model-free, nonlinear extension of Granger causality. Like Granger causality, it quantifies the predictive power of the past of a source signal toward the future of a target signal, beyond the predictive power of the past of the target signal itself (Lee et al., 2015). In simpler terms, TE provides a way to assess the directed influence or causal relationship between two time series. Unlike Granger causality, TE does not presuppose any relationship between these signals. Symbolizing TE involves substituting the values in both the source and target signal vector components with ranked values (i.e., symbols), which eliminates some of the parameter selection inherent to TE, making the method more robust and computationally efficient (Staniek and Lehnertz, 2008). The normalization of STE removes bias introduced by signal characteristics of the source signal and autocorrelation within the target signal, making STE values comparable across different pairs of time series (Lee et al., 2015).

NSTE was computed between each dyad’s kinematic source signals, where the combination of participant hands used for the input time series was identical to that used to calculate the MCV. This enabled us to directly compare the undirected correlation measure of coordination (i.e., MCV) with the directed, information theory-based metric (i.e., NSTE) to assess its added value to capturing spontaneous interactivity. We computed NSTE for all rated moments of high and low coordination, using an embedding dimension of *m* = 3, a window size of 2 s, a window step size of 0.5 s, and varying tau systematically to span a prediction range of 100 ms (*τ* = 6) to 250 ms (*τ* = 15). Parameter selection occurred by identifying three moments with clear lead-follow relationships through visual inspection of the video recordings and selecting the parameters that maximized NSTE values during the moments and minimized them outside the moments. We then calculated the asymmetry of NSTE values in each direction (i.e., NSTE_A → B_ and NSTE_B → A_) to assess the dominant direction of information flow for each time step, as follows:


$$\mathrm{Asymmetr}y_{A\to B}=\frac{NSTE_{A\to B}-NSTE_{B\to A}}{NSTE_{A\to B}+NSTE_{B\to A}}\varepsilon \left[-1,1\right]$$

Thus, if asymmetry_A → B_ has a positive value, the information flow from A to B is dominant, and vice versa for a negative value.

#### 2.5.4 Subjective reports

In this study’s third research question, we ask: what is the relationship between detected moments of high coordination and the relational dimensions of the dancers’ experiences of moving together? To address this, we identified moments of rated high coordination that co-occurred (i.e., +/− 180 s total) with segments marked by the participants (see Section 2.3.3). From these segments, we extracted participants’ perceptions of coordination, role, and connection with their partner as well as any commentary, and examined these subjective reports alongside measures of coordination for patterns. Participant reports about attention and movement intentionality (questions 2c and 2d) were not considered in the current analysis as the goal of this analysis was to investigate the relational dimensions of moving together rather than each individual’s cognitive processes.

## 3 Results

Human raters of the videotaped improvisations identified 37 moments of sustained high or low coordination across five unique dyads. Of these 37 moments, 24 were rated as high coordination, while 13 were rated as low, with substantial inter-rater reliability (Fleiss’ kappa = 0.68). High coordination moments were more likely to occur when both participants had their eyes open (16/24). Moments of low coordination were more evenly distributed across the different perceptual conditions.

**Figure 4:**
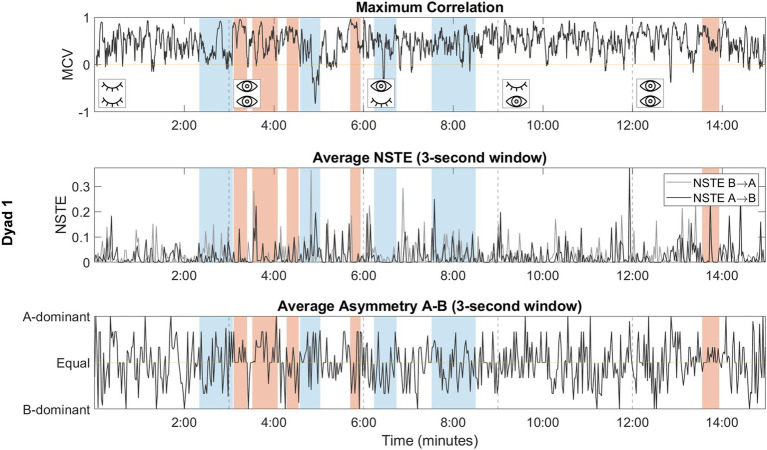
Overview of each coordination measure derived from a single session (dyad 1). Dotted lines delineate the boundaries of each perceptual condition. Moments of rated high coordination are denoted by red bars, while moments of rated low coordination are denoted by blue bars. To enhance signal clarity, the NSTE and asymmetry plots have been smoothed using non-overlapping 3-s windows.

Figure 4 provides an overview of all measures of interpersonal coordination calculated in our analysis across a full improvisation task, with human-rated moments of high coordination (red) and low coordination (blue) overlayed.

### 3.1 Moments of interpersonal coordination can be accurately detected by maximum correlation vectors

The MCV classified moments of high and low coordination with an overall accuracy of 80.56%, a precision of 65.52%, and a sensitivity of 79.17%. The current algorithm is biased toward the classification of high coordination; considering the study’s focus on moments of high coordination as potential indicators of social connection, a preference for over-detection is acceptable, as it reduces the likelihood of overlooking moments of connection that would otherwise go undetected.

**Figure 5:**
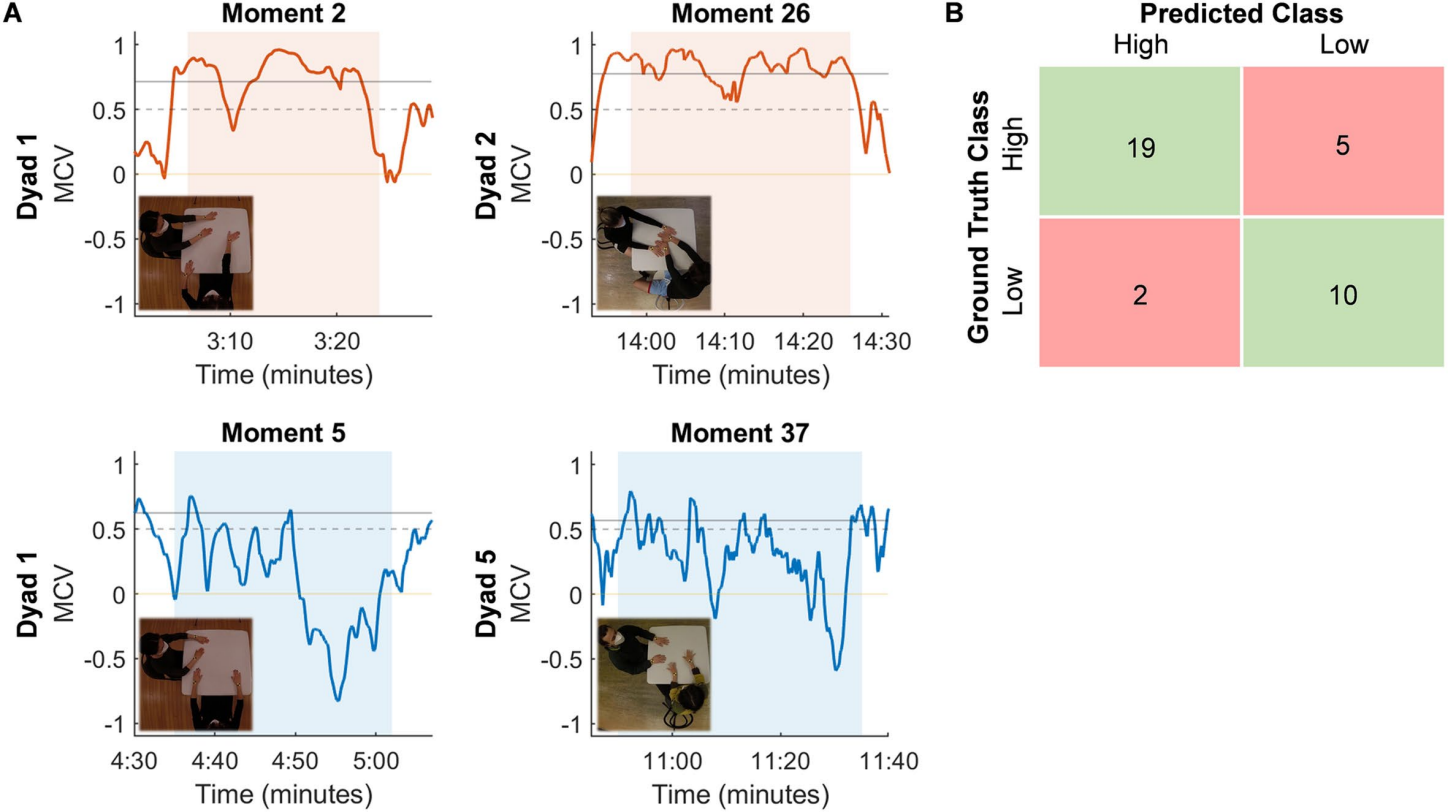
**(A)** Four representative moments illustrating true positive and true negative instances. For each moment, the maximum correlation vector is depicted alongside a vignette of the corresponding segment of interaction. High coordination moments are highlighted by orange panels and low coordination moments by blue panels. Solid grey lines denote the unique significance threshold for each moment while the dotted lines represent the classifier threshold *y* = 0.5. **(B)** Confusion matrix summarizing the results of classification.

We evaluate and describe a representative selection of four moments with respect to the dynamics observed during moments of high and low coordination (Figure 5). Moments 2 and 26 were human-rated as high coordination and show sustained high values of the MCV, which decreases within a few seconds at the boundaries of the moment. The hand movements of both dancers show comparable speeds during these moments. Both moments include brief periods where the MCV drops below the significance and classifier thresholds; references to the corresponding moments in the videos confirm that these periods coincide with observable decreases in hand coordination.

Moments 5 and 37 were human-rated as low coordination and show MCV values that remain predominantly below the significance and classifier thresholds. Additionally, the MCV exhibits high variability and is not significantly different at the boundaries of these moments in comparison to patterns within the moment. Moment 37 involved fast hand movements across both dancers, reflected in the higher frequency and variability of the MCV; in contrast, Moment 5 involved slower and smoother hand movements, reflected in the lower frequencies of the MCV.

### 3.2 NSTE complements correlation metrics by revealing the dynamics of directed influence during moments of interpersonal coordination

**Figure 6:**
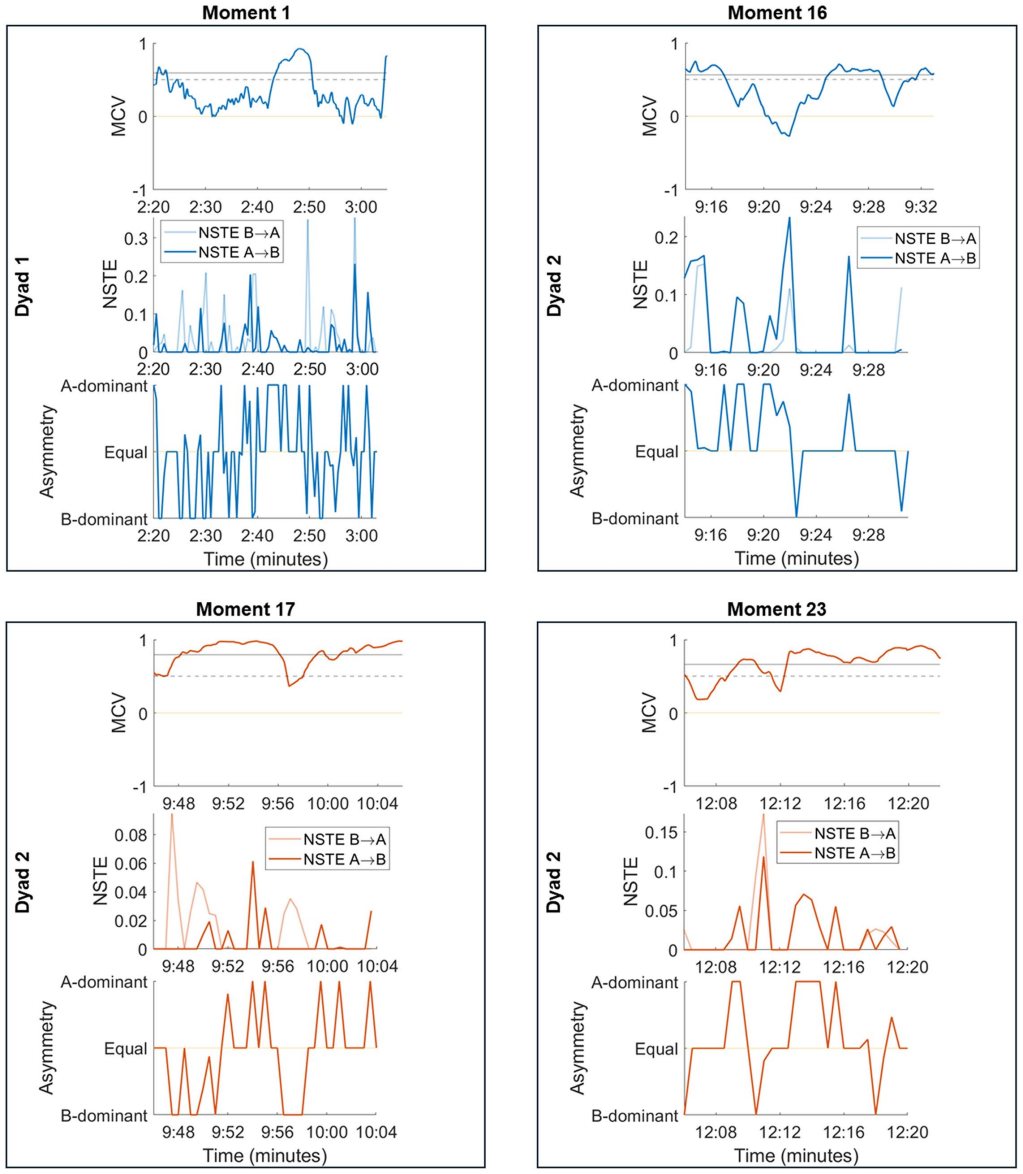
MCV, NSTE, and NSTE asymmetry from representative moments of high coordination (orange) and low coordination (blue). MCV significance threshold is indicated by the dotted line, while the classifier threshold is indicated by the solid line.

The magnitude and relative dominance of the causal relationship between dancers provide a complementary perspective on moments of high and low interpersonal coordination beyond correlational metrics alone. We present MCV and NSTE metrics across four representative moments of low and high coordination to illustrate the value added by considering directional measures (Figure 6).

Moment 1 was classified as a low coordination moment by both human raters and the MCV algorithm. NSTE analysis reveals multiple transient moments of casual influence between dancers, who alternate between leading and following. We visually validated several NSTE peaks with the video recording, confirming that they occurred during leader-follower interactions between the two dancers (e.g., dancer B pushing dancer A at 2:49, reflected in a high MCV and a peak in NSTE_B → A_). In this example, the NSTE magnitude during a transient MCV peak revealed the direction of influence between the dancers. Moment 16 was also classified as a low coordination moment. The MCV falls below zero at approximately 9:22; visual inspection of the corresponding video recording shows a call-response interaction between the dancers where dancer A briefly pauses followed by a similar pause from dancer B. At the beginning of this moment, dancer A leads B; at the end, the roles have reversed, reflected in the NSTE asymmetry.

Moment 17 and 23 were both classified as high coordination moments. Moment 17 consisted of subtle pushing between the dancers, corresponding to the back-and-forth dynamics in the NSTE asymmetry. In Moment 23, around 12:11, the dancers begin to move together in a circular motion, connected by a single fingertip. Although it is not clear from the video which dancer is leading, the NSTE metrics show dominant information flow from dancer A to dancer B, with a brief reversal of roles around 12:18.

### 3.3 Detected moments of high coordination reflect a one-to-many relationship with subjective experience

**Figure 7:**
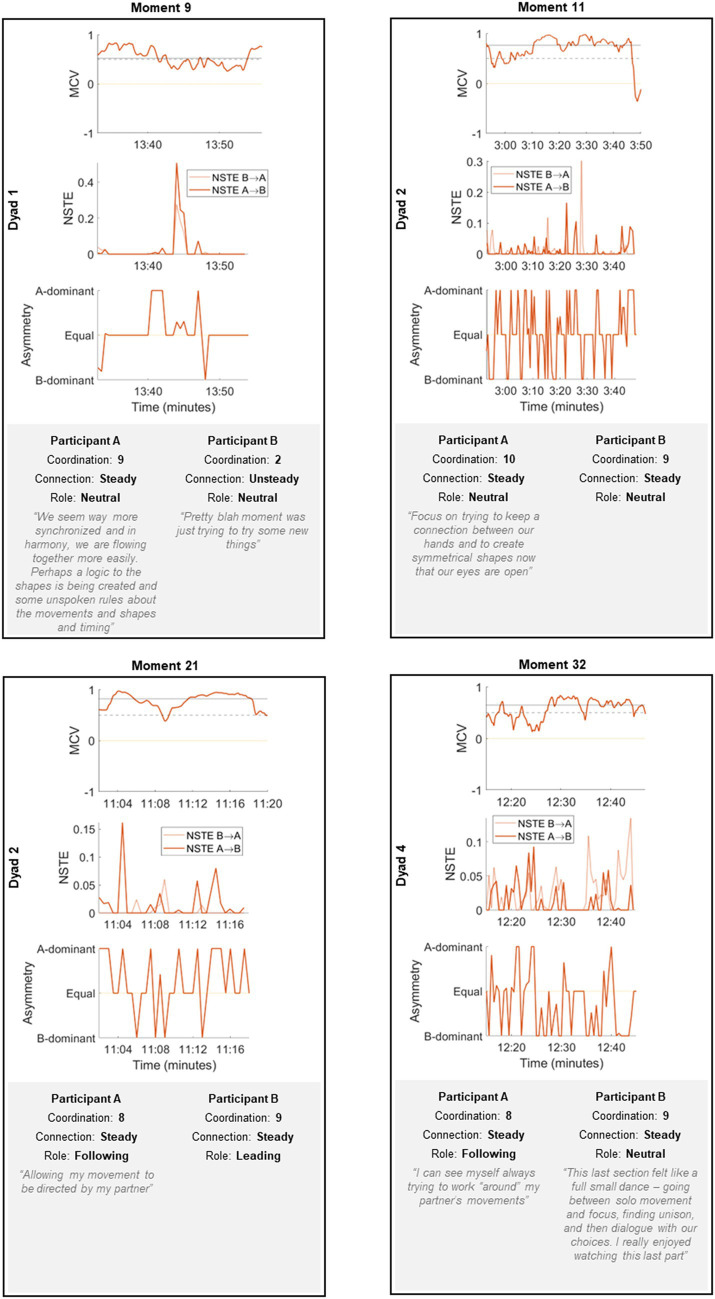
Cross-section of MCV, NSTE, and NSTE asymmetry from four moments of high coordination sufficiently overlapping with participants’ segments, alongside commentary provided by participants.

Participant comments on segments that overlapped with classified moments of high interpersonal coordination were associated with a range of experiences (Figure 7). The majority of participants reported a subjective experience of high coordination with their partner during these moments; subjective experiences of leading/following did not always correspond with the partner, nor with the NSTE metrics.

In Moment 9, dancer A perceived the moment as highly generative, coordinated, and connected, whereas dancer B viewed it as relatively dull, uncoordinated, and disconnected. The MCV during this moment fluctuated dynamically near the significance threshold, indicating inconsistency in the temporal alignment of their movements and generally lower levels of coordination, consistent with the experiences of dancer B. The NSTE metrics show near-zero causal influence between dancers for most of the moment, with the exception of several seconds when dancer A leads the movement of dancer B; this may be reflected in dancer A’s subjective report of the experience of the moment.

There is strong coherence between the participants’ reported experiences and coordination ratings in Moments 11 and 21. In Moment 11, the MCV is high across the duration of most of the moment, indicating heightened coordination between dancers. The NSTE metrics are very dynamic. Consistent with neither participant perceiving themselves as leading or following, the asymmetry between dancer A and B appears to be fairly balanced, with dancer A showing slightly more influence. Dancer A reported that the dyad was focusing on symmetry and creating shapes in this moment; the dynamic character of this moment likely reflects the many micro-adjustments from both participants needed to accomplish this. The MCV in Moment 21 is also high and particularly sustained, indicative of fluid coordination. The NSTE metrics show a sustained influence of dancer A on dancer B. Notably, this is in contrast to the dancers’ subjective reports of the moment, where both participants reported that dancer A was following dancer B.

In Moment 32, participants reported high levels of coordination and a consistent connection with their partner. Dancer A perceived themselves as following their partner, while dancer B was neutral about their role within the moment. This was consistent with the results of the NSTE analysis, which showed dominant information flow from dancer B to A for the majority of the moment.

## 4 Discussion

This study presents a novel method for capturing spontaneous interactivity and characterizing the time-varying dynamics of coordination patterns within interpersonal interactions. We employ three metrics based on movement correlation, information theory, and subjective report to capture a cross-section of complementary perspectives on the emergence of high coordination moments between two freely improvising dancers. Our movement correlation metric—the maximum correlation vector (MCV)—classified moments of high and low coordination with an accuracy of 80%, illustrating the efficacy of automatically detecting these moments from video recordings using correlation-based measures. Our application of an information theory metric—normalized symbolic transfer entropy (NSTE)—to measure the direction of influence between dancers captured the causal influence between interacting partners. This is the first study to use NSTE on kinematic data and demonstrates the potential of this metric to reveal subtler dynamics of interpersonal interaction. Finally, we demonstrate a complex relationship between objective coordination measures and the relational dimensions of subjective experience, highlighting the need for principled approaches to the collection of first-person experiential data. Altogether, this study offers a novel framework for investigating the dynamics of interactions and the interpersonal domain, providing a strong platform for future research on the effects of moving together.

We define interpersonal coordination as the temporal alignment of motor behavior between two or more individuals. This includes instances where participants are temporally coupled while not necessarily rhythmically entrained or precisely matching each other’s behavior. Interpersonal coordination is canonically described as “the degree to which the behaviors in an interaction are non-random, patterned or synchronized in both timing [and] form” (Bernieri and Rosenthal, 1991). In our study, interpersonal coordination has reduced dependence on form, and less emphasis is placed on the specific physical configurations and trajectories of movement (Smykovskyi et al., 2022; Koul et al., 2023a). Specifically, the MCV is calculated from speed rather than the velocity of the dancers’ hands: this captures the temporal coherence of movement while accommodating diversity in movement form. Additionally, MCV is high during moments of joint stillness, as stillness does not necessarily imply a lack of coordination. The MCV is especially valuable in the context of freely improvised movement, where the lack of periodicity renders popular phase-based approaches (e.g., evaluating in-phase and anti-phase coordination) unsuitable (Marsh et al., 2009; Miles et al., 2017; Schmidt and Richardson, 2008). The high accuracy (~80%) of the MCV in detecting moments of high and low coordination demonstrates its alignment with both our conceptual understanding and visual perception of interpersonal coordination. This correlation-based metric has strong potential as the basis of an automatic system for assessing time-varying dynamics of coordination in the context of dance.

To date, much research on interpersonal coordination within dance has relied on phase-based measures to assess synchrony in oscillatory movements among individuals, with two notable exceptions. Boker and Rotondo studied non-expert movers who alternated between leading and following their partner in short free-form dance trials. Interpersonal coordination was measured using time-varying correlations between the total displacement of participants, which was calculated by averaging velocity signals from all tracked body parts across time (Boker and Rotondo, 2002). Brown and Meulenbroek explored interpersonal coordination in choreographed dance by correlating the acceleration of vertical hand movement among trained dancers (Brown and Meulenbroek, 2016). Our study improves upon these correlation-based measures in several important ways. First, Boker and Rotondo’s approach of averaging velocity across all body parts obscures fine-grained information about the spatial similarity of movement; our study tracks coordinated moments between the same body part between dancers, preserving more nuance and detail. Second, Brown and Meulenbroek’s method captures spatial and temporal alignment of the dancers, but it is only suitable for scenarios where participants perform identical actions. In contrast, our method is able to capture the complex dynamics of breaking and building symmetry that emerge in improvised versus choreographed dance. Furthermore, our study provides a metric that can be applied in the absence of all external cues for synchronization between dancers. Boker and Meulenbroek’s study involved an external beat to which participants were dancing; their results are limited to rhythmically motivated movement coordination, rather than spontaneous intrinsically motivated coordination. Despite the movement being free-form, the coordination observed in their study cannot be solely attributed to the co-creation of participants. Brown and Meulenbroek’s study involves choreography—a dance context with tasks designed to establish shared performance goals. This represents markedly different interaction dynamics compared to improvised dance settings. During choreography, the emerging interpersonal coordination may be more appropriately described as joint action—interpersonal behavior that is unified in a common desired outcome (Keller et al., 2014). Although joint action may be intricate, such as in ensemble dance, it represents a different kind of complexity when contrasted with the spontaneously co-created movement coordination observed in free improvisation. This approach taken in our study allows for the interpretation of high correlations as spontaneous coordination independent of external time-keeping cues, providing a clearer understanding of interpersonal coordination dynamics.

Our study is the first to apply NSTE to kinematic data. To date, NSTE has primarily been applied to intrapersonal physiological recordings. Previous studies have used it to measure the direction of information flow within the brain (Lee et al., 2013; Borjigin et al., 2013), and to investigate causal relationships amongst different types of intrapersonal physiological recordings, such as between brain and heart signals (Li et al., 2015). Some studies have used NSTE to examine directed coupling relationships between individuals to assess interpersonal physiological synchrony (Fu et al., 2021). Our study’s application of NSTE to interpersonal kinematic data offers several advantages over other measures of directed influence, such as Granger causality (Granger, 1969), dynamical causal modeling (Friston et al., 2003), and (windowed) cross-lagged correlations. While Granger Causality has been used in studies of group coordination and leadership dynamics in musical ensembles (e.g., Hilt et al., 2019; Sabharwal et al., 2024), NSTE is particularly well-suited for contexts like free improvisation, where individuals’ roles constantly shift. In contrast to the more structured dynamics of music performance, where leader-follower roles are often clearly defined, free improvisation involves spontaneous and flexible exchanges of influence. NSTE’s ability to detect non-linear relationships and provide a model-free estimation of connectivity makes it ideal for capturing these dynamics (Lee et al., 2015). It is also computationally efficient and requires fewer *a priori* parameter selections than other methods. This measure is thus highly versatile and particularly well-suited for analyzing kinematic signals from naturalistic interactions, which are often non-stationary and non-linearly related.

In line with Varni et al. (2022), who examined the effect of leadership changes on entrainment in music ensembles, our work similarly aims to be able to describe how shifts in leader-follower roles affect coordination. Varni et al. (2022) concept of “soft entrainment”—where synchrony fluctuates rather than remains fixed—is consistent with our view of the flexible, adaptive nature of group coordination during free improvisation in dance. Furthermore, the work by Sabharwal et al. (2024) on the directionality of influence in musical performance complements our observations of leadership dynamics. Their findings suggest that greater directionality occurs when clear leader-follower roles are established, while co-creation, and thus improvisation in dance, lead to more mutual, less directional interaction. This aligns with our results, where periods of spontaneous collaboration showed fluid exchanges of influence, supporting the notion of co-created movement.

A major strength of this study is its use of mixed methods, with complementary quantitative and qualitative levels of description of intersubjectivity, with each level providing different perspectives on the nature of interactions as complex systems. The first level treats the interaction as the unit of analysis, measuring interpersonal coordination through correlations in participant movement and quantifying the collective dynamics as an emergent property of the interaction system. The second level captures the interactions *between* components of the interaction system. The NSTE analysis quantifies the contribution and directionality of both components simultaneously. Thus, this measure is a proxy for how each interactor contributes to constructing the interaction at any given point in time, providing a richer description of the co-constructive nature of collective movement. The third level qualitatively captures our participants’ subjective experience of moving together, focusing on the relational dimensions of this experience such as the sense of connectedness between participants. This multi-measure approach, which links multiple levels of description, offers distinct insights into the dynamics of the interaction and the ability of these levels to mutually inform each other. Mixed-method approaches to studying intersubjectivity have been advocated in multiple theoretical frameworks, such as *participatory sense-making* (De Jaegher and Di Paolo, 2007). Participatory sense-making is a theory of social cognition stemming from the enactive tradition of philosophy of mind that centers the *interaction process* in the query of intersubjectivity. It asserts that social understanding transcends mere individual mentalizing, emphasizing our mutual participation in meaning-making. Participatory sense-making underscores how interactions with the environment and others shape our understanding and aims to elucidate the role of interaction processes in sense-making experiences. By examining interaction dynamics across various levels of description without explanatory reduction from one level to the next, our approach respects the ontological status of the interaction process posited by influential philosophical literature on intersubjectivity. This approach allows a comprehensive exploration of the relationship between interaction dynamics, such as interpersonal coordination, and the relational dimensions of our social experiences. The methodological framework for investigating intersubjectivity used in this study can be used in future work to explore research questions related to participatory sense-making and as a tool for assessing specific outcomes of dance and their interactive underpinnings.

The results of this study need to be interpreted in light of several limitations. First, we do not explore moments of low interpersonal coordination or the transitions into and out of moments of high coordination. These transitions have been posited as crucial investigation points to better grasp the relationship between interpersonal coordination and subjective experience (Di Paolo and De Jaegher, 2012). To address this gap, future research should prioritize acquiring more nuanced first-person data, potentially through the use of phenomenological interviews, which aim to explicate the fine-grained details of subjective experiences (Olivares et al., 2015). Second, our approach misses spontaneously emerging interaction dynamics other than temporal coordination, such as call-and-response and mimicry. Such behaviours are fundamental features of dynamic interpersonal interactions and are often intuitively considered forms of coordination; the conceptual and operational definitions used in this study remain limited in scope.

## 5 Conclusion

In this study, we developed a novel approach to capturing interpersonal coordination within the context of dance improvisation. By integrating correlation-based measures of movement speed, novel applications of NSTE to kinematic data, and subjective experiential data, our approach offers a multi-dimensional lens to capture the intricate dynamics of co-creative interactions. Our findings underscore the effectiveness of correlation-based measures in detecting coordination patterns and highlight the utility of NSTE in revealing the directionality of influence between interactors. Furthermore, our definition of interpersonal coordination, emphasizing temporal similarity over spatial similarity, expands our understanding of coordination, particularly in the context of freely improvised dance. Our study advances the tools available for examining the intricate relationship between interpersonal coordination and subjective experiences in dance. Future research should aim to explore the transitions between moments of coordination, examine complementary interaction dynamics, and incorporate more comprehensive first-person experiential data. With dance’s potential to enhance wellbeing and interpersonal connection, our methodological framework can pave the way for further investigations and applications in therapeutic and creative contexts.

## Data Availability

The datasets presented in this study can be found in online repositories. The names of the repository/repositories and accession number(s) can be found at: https://osf.io/6hz35/.
